# Supplementary material for: Impact of Lymphovascular Invasion on Prognosis in the Patients with Bladder Cancer—Comparison of Transurethral Resection and Radical Cystectomy
Source: Diagnostics (Basel). 2021 Feb 4;11(2):244. doi: 10.3390/diagnostics11020244 (PMC7915441; doi:10.3390/diagnostics11020244)
Supplement: Supplementary file 1 [file diagnostics-11-00244-s001.zip › diagnostics-1025752-supplementary/Supplementary XML.docx]

**Table S1.** Clinical and pathological characteristics of patients at first TURBT.

| Characteristics *n* = 216 | |
| --- | --- |
| Age, years (±SD) | 71.3 (±9.3) |
| Males, *n* (%) | 172 (79.6) |
| Females, *n* (%) | 44 (20.4) |
| Smoking status, *n* (%) | 129 (59.7) |
| Clinical stage, *n* (%) |  |
| stage 0a, I | 163 (75.5) |
| stage II | 30 (13.9) |
| stage III | 10 (4.6) |
| stage IV | 14 (6.5) |
| Multiple tumors, *n* (%) | 98 (45.4) |
| Non papillary, *n* (%) | 26 (12.0) |
| Sessile, *n* (%) | 120 (55.6) |
| Pathological T stage, *n* (%) |  |
| pTa | 100 (46.3) |
| pT1 | 76 (35.2) |
| ≥pT2 | 40 (18.5) |
| Presence of G3, *n* (%) | 94 (43.5) |
| CIS+, *n* (%) | 21 (9.7) |
| LVI+, *n* (%) | 49 (22.7) |
| histological variant, *n* (%) | 22 (10.2) |
| intravesical instillation after TURBT (BCG) | 47 (21.8) |

TURBT: transurethral resection of bladder tumor; SD: standard deviation; CIS: carcinoma in situ; LVI: lymphovascular invasion.

**Table S2.** Clinical and pathological characteristics of patients who underwent RC.

| Characteristics *n* = 64 | |
| --- | --- |
| Age, years (±SD) | 68.2 (±7.9) |
| Males, *n* (%) | 51 (79.7) |
| Females, *n* (%) | 13 (20.3) |
| Neoadjuvant chemotherapy, *n* (%) | 20 (31.3) |
| Pathological T stage, *n* (%) |  |
| pT0 | 19 (29.7) |
| pTa, Tis | 3 (4.7) |
| pT1 | 10 (15.6) |
| pT2 | 17 (26.6) |
| pT3 | 12 (18.8) |
| pT4 | 3 (4.7) |
| Lymph node metastasis, *n* (%) | 13 (20.3) |
| Distance metastasis, *n* (%) | 1 (1.6) |
| Presence of G3, *n* (%) | 34 (53.1) |
| CIS+, *n* (%) | 7 (10.9) |
| LVI+, *n* (%) | 21 (32.8) |
| histological variant, *n* (%) | 6 (9.4) |
| intravesical instillation before RC (BCG), *n* (%) | 8 (12.5) |

RC: radical cystectomy; CIS: carcinoma in situ; LVI: lymphovascular invasion; TURBT: transurethral resection of bladder tumor; RC: radical cystectomy.

**Table S3.** Clinical and pathological characteristics of patients receiving or not receiving NAC.

|  | NAC+  (*n* = 15) | NAC−  (*n* = 35) | *p*-value |
| --- | --- | --- | --- |
| Age, years (range) | 69 (43–79) | 70 (52–82) | 0.511 |
| Males, *n* (%) | 13 (86.7%) | 27 (77.1%) | 0.440 |
| Females, *n* (%) | 2 (13.3%) | 8 (22.9%) |  |
| Clinical stage, *n* (%) |  |  | 0.006 |
| stage 0a, I, II | 8 (53.3%) | 31 (88.6%) |  |
| stage III, IV | 7 (46.7%) | 4 (11.4%) |  |
| LVI+ at first TURBT | 14 (93.3%) | 17 (48.6%) | 0.003 |
| RC | | | |
| Pathological T stage, *n* (%) |  |  | 0.044 |
| <pT3 | 9 (60.0%) | 30 (85.7%) |  |
| ≥pT3 | 6 (40.0%) | 5 (14.3%) |  |
| Lymph node metastasis, *n* (%) | 4 (40.0%) | 5 (14.3) | 0.088 |
| Presence of G3, *n* (%) | 9 (60%) | 16 (45.7%) | 0.355 |
| CIS+, *n* (%) | 2 (13.3%) | 3 (8.6%) | 0.607 |
| LVI+, *n* (%) | 6 (40.0%) | 9 (25.7%) | 0.312 |

**Table S4.** Changes in LVI with or without NAC in LVI-positive cases at first TURBT.

|  | **First TURBT LVI+** | | ***p*-value** |
| --- | --- | --- | --- |
|  | **NAC+**  **(*n* = 14)** | **NAC−**  **(*n* = 17)** |  |
| **RC LVI-** | 8 (57.1%) | 13 (76.5%) | 0.252 |
| **RC LVI+** | 6 (42.9%) | 4 (23.5%) |  |

LVI: lymphovascular invasion; NAC: neoadjuvant chemotherapy; RC: radical cystectomy; TURBT: transurethral resection of bladder tumor.
