# Supplementary figures and images for: Impact of Lymphovascular Invasion on Prognosis in the Patients with Bladder Cancer—Comparison of Transurethral Resection and Radical Cystectomy
Source: Diagnostics (Basel). 2021 Feb 4;11(2):244. doi: 10.3390/diagnostics11020244 (PMC7915441; doi:10.3390/diagnostics11020244)

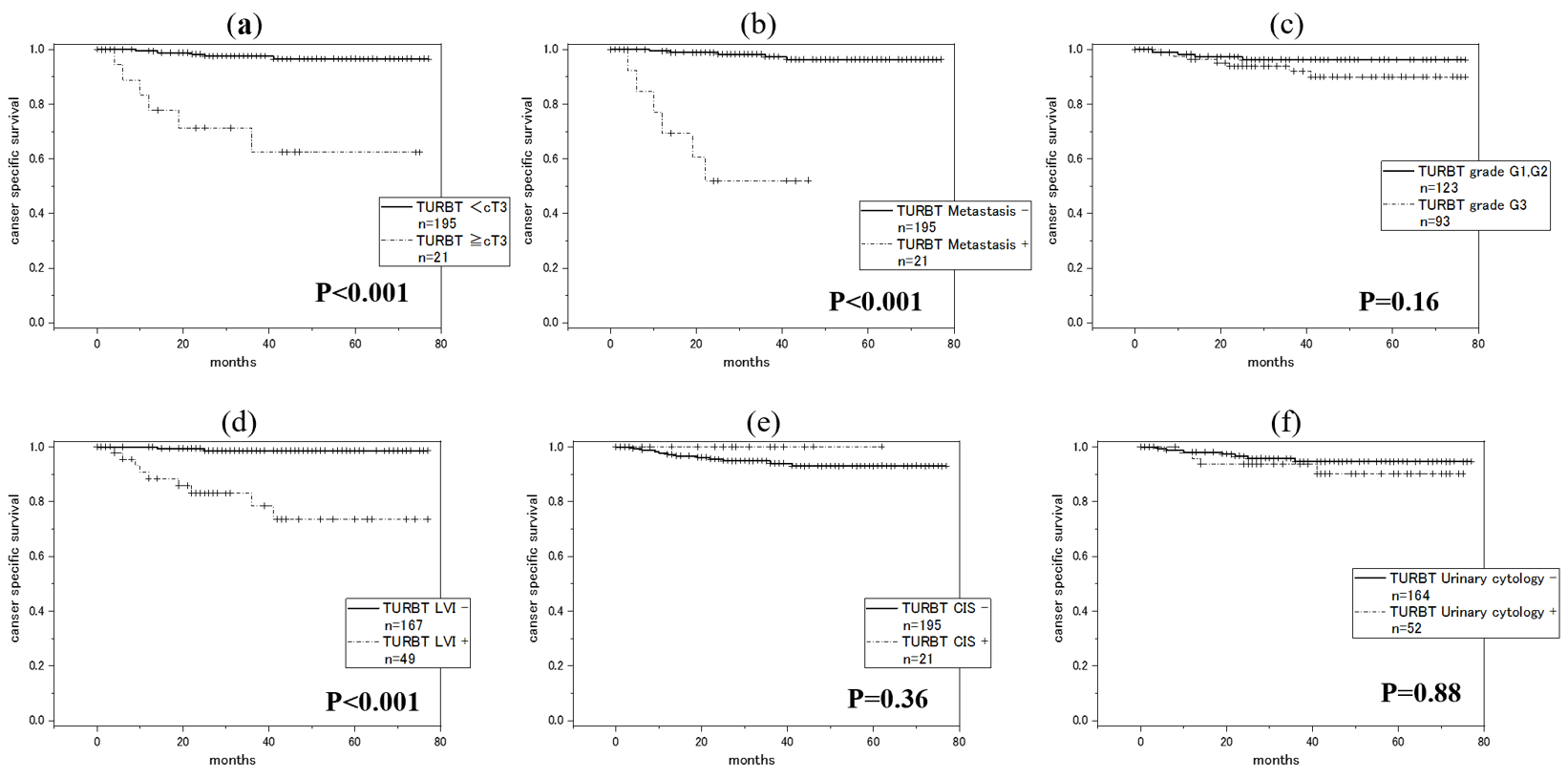

Supplement: Supplementary file 1 [file diagnostics-11-00244-s001.zip › diagnostics-1025752-supplementary/supplementary Figure1 (2).tif]

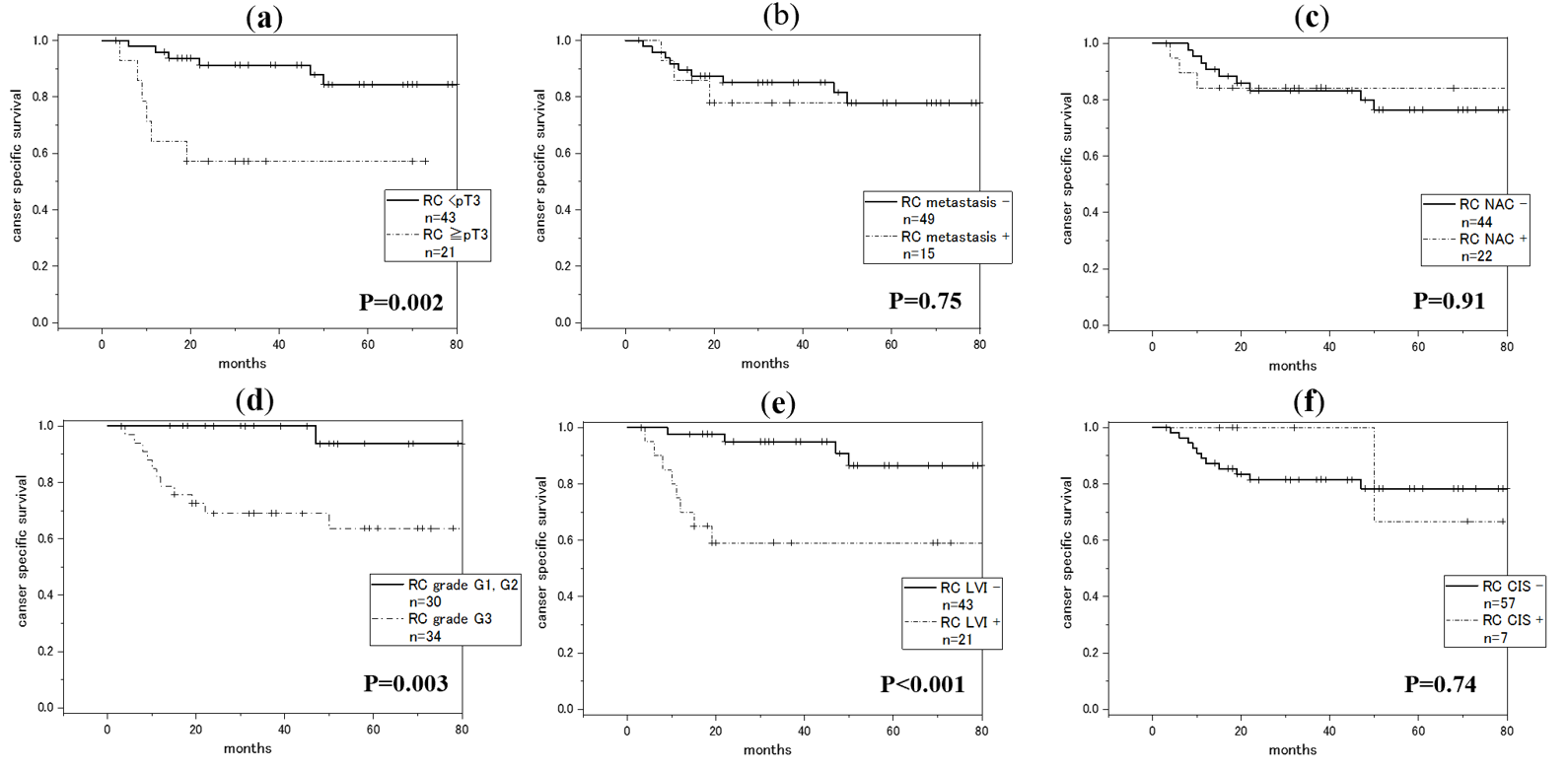

Supplement: Supplementary file 1 [file diagnostics-11-00244-s001.zip › diagnostics-1025752-supplementary/SupplementaryFigures2 (1).tif]
